# Heritability of obsessive–compulsive trait dimensions in youth from the general population

**DOI:** 10.1038/s41398-018-0249-9

**Published:** 2018-09-18

**Authors:** Christie L. Burton, Laura S. Park, Elizabeth C. Corfield, Nadine Forget-Dubois, Annie Dupuis, Vanessa M. Sinopoli, Janet Shan, Tara Goodale, S.-M. Shaheen, Jennifer Crosbie, Russell J. Schachar, Paul D. Arnold

**Affiliations:** 1 Neurosciences and Mental Health, Hospital for Sick Children, Toronto, Canada; 2 Genetics and Genome Biology, Hospital for Sick Children, Toronto, Canada; 3 Institute of Medical Science, University of Toronto, Toronto, Canada; 4 Department of Psychiatry, University of Toronto, Toronto, Canada; 5 École de Psychologie, Université Laval, Quebec City, Canada; 6 Clinical Research Services, Hospital for Sick Children, Toronto, Canada; 7 Dalla Lana School of Public Health, University of Toronto, Toronto, Canada; 8 Mathison Centre for Mental Health Research and Education, Departments of Psychiatry and Hotchkiss Brain Institute, Cumming School of Medicine, University of Calgary, Calgary, Canada

## Abstract

Obsessive–compulsive disorder (OCD) is a heritable childhood-onset psychiatric disorder that may represent the extreme of obsessive–compulsive (OC) traits that are widespread in the general population. We report the heritability of the Toronto Obsessive–Compulsive Scale (TOCS), a new measure designed to assess the complete range of OC traits in youth. We also examined the dimensional nature of the TOCS and the degree to which genetic effects are unique or shared between dimensions. OC traits were measured using the TOCS in 16,718 youth (6–18 years) at a science museum. We conducted a factor analysis to identify OC trait dimensions. We used univariate and multivariate twin models to estimate the heritability of OC trait dimensions in a subset of twins (220 pairs). Six OC dimensions were identified: Cleaning/Contamination, Symmetry/Ordering, Rumination, Superstition, Counting/Checking, and Hoarding. The TOCS total score (74%) and each OC dimension was heritable (30–77%). Hoarding was not highly correlated with other OC dimensions, but did share genetic effects. Shared genetics accounted for most of the shared variance among dimensions, whereas unique environment accounted for the majority of dimension-specific variance. One exception was Hoarding, which had considerable unique genetic factors. A latent trait did not account for the shared variance between dimensions. In conclusion, OC traits and individual OC dimensions were heritable, although the degree of shared and dimension-specific etiological factors varied by dimension. The TOCS may be informative for genetic research of OC traits in youth. Genetic research of OC traits should consider both OC dimension and total trait scores.

## Introduction

The role of genetics in the etiology of obsessive–compulsive disorder (OCD) is well established^1^. OCD is familial and heritable^2–5^. In children and adolescents, genetic effects account for most of the variance (heritability estimates of 45–65%)^6^ and the stability of symptoms across development^7^, with common environment only playing a role in early adolescence^8,9^. However, there are few replicated genetic risk variants for OCD. Gene discovery has been hampered by relatively small samples, which international consortia are working to overcome^10^.

Exclusive reliance on OCD diagnosis and clinic samples may be another reason for slow progress. Diagnoses are useful in clinical practice, but in genetic research, could obscure phenotypic and genetic heterogeneity, hide variation in symptom severity among cases, and miss sub-threshold OCD cases^11^. Clinical samples are slow and expensive to collect. Alternatively, if OCD represents an extreme of obsessive–compulsive (OC) traits widely distributed in the population, then genetic research could focus on quantitative OC trait measures. A quantitative trait-based measure assesses the full range of OC traits (e.g., from extreme difficulty, to complete ease, discarding useless objects) to capture all their variance, which could boost power for genetic studies^11,12^, especially in general population samples.

Existing OCD scales can generate quantitative scores by summing the number of symptoms exhibited by an individual. However, symptom counts typically generate J-shaped distributions in population samples because most people have few or no OC symptoms. These kind of scores are suboptimal for quantitative analyses.

Another reason for slow progress in genetic discovery might be the apparent phenotypic heterogeneity in OCD. In adults and youth, OC symptoms generally cluster into four dimensions: symmetry, forbidden thoughts/checking, cleaning, and hoarding^13,14^, indicating considerable phenotypic heterogeneity. Twin studies in adults indicate that each of these dimensions is heritable with genetic influences that are shared among dimensions and unique to each dimension^15,16^, suggesting that phenotypic heterogeneity reflects genetic heterogeneity. In youth, the heritability of OC traits and dimensions is unclear^17^, particularly the degree to which individual dimensions are mediated by unique and shared genetic influences. Hoarding is considered a distinct dimension in adults and youth and is classified as its own disorder in the Diagnostic and Statistical Manual of Mental Disorders, 5th edition (DSM-5^18^). Twin studies in adults suggest that hoarding shares genetic influences with other OC dimensions^15,19,20^. In youth, it is not known whether hoarding shares genetic risk with other OC dimensions. The present study aimed to uncover the degree of shared and unique genetic effects on OC dimensions in youth.

We developed the Toronto Obsessive–Compulsive Scale (TOCS^21^) to measure widely distributed OC traits in youth and to capture OC dimensions. Each item on the TOCS queries whether a given OC symptom occurs far less often (lower extreme), an average amount of time, or far more often (upper extreme) in the child than in typically developing peers. Questionnaires of this type are designed to generate scores that are widely and more normally distributed in the general population compared with the J-shaped distributions found with typical symptom-based measures (see Supplemental Fig. 1 for TOCS distributions). By including both upper and lower extremes of OC traits, the TOCS can distinguish between the absence of OC symptoms (typically coded as “0” in symptom count-based scales) and the lower extreme.

We tested whether (1) the TOCS captured OC dimensions, (2) if these dimensions were heritable as well as co-heritable, (3) if these dimensions had shared and unique etiological factors, and (4) whether shared variance between the dimensions was best explained by a common pathway (single global latent trait) or independent pathway model. To address these questions, we factor analyzed the TOCS in a population-based sample (*n* = 16,718) of youth to identify OC dimensions. We used structural equation modeling in 220 twin pairs to examine the heritability of the individual dimensions, their co-heritability and test the fit of the independent and common pathway models. Current evidence indicates that the best fitting model is unclear in adults^15,16^ and unknown in youth. The expectation was that if a common pathway model fit best, then shared etiological factors were mediated by an underlying latent trait, whereas if an independent pathway model fit best, then shared etiological factors influenced dimensions directly. If the variance shared by multiple dimensions is captured by a global latent trait, research designs should focus on that latent trait. Conversely, if unique genetic factors influence each OC trait dimension directly, research designs should focus on the individual OC dimensions.

## Subjects and methods

### Sample and design

Our sample included 16,718 participants with complete data (mean age 11.1 years (standard deviation; SD 2.8); 50.5% male) from the 17,263 youth (6–18 years of age) recruited at the Ontario Science Centre, a local science museum in Toronto, Canada. Informed consent, and assent where applicable, was obtained and the protocol was approved by The Hospital for Sick Children Research Ethics Board. We collected behavioral information about the participants from themselves if they were thought to be capable of self-reporting (18.2%) or from their parents (81.8%). Participants provided a saliva DNA sample using 2 mL Oragene^®^ kits (DNA Genotek, Ottawa, Canada).

### Measures

A computerized, English questionnaire covered demographics, medical history, and two measures of OC traits and symptoms. The TOCS had 21 items scored on a scale of − 3 to + 3 (− 3 = far less often than average; − 2 = less often than average; − 1 = slightly less often than average; 0 = average amount of time; 1 = slightly more often than average; 2 = more often than average; and 3 = far more often than average). The TOCS has been shown to have high internal consistency (Cronbach’s *σ* = 0.94) and to discriminate between an OCD measure (the Obsessive–Compulsive Scale of the Child Behavior Checklist (CBCL-OCS^22,23^) Spearman correlation = 0.5) and an ADHD measure (the Strengths and Weaknesses of ADHD Symptoms and Normal Behavior scale (SWAN^24^), Spearman’s correlation = 0.02)^21^. Because the TOCS total score (sum of all 21 items; range: − 63 to 63) was significantly associated with age, gender and respondent (*p* < 0.05), we created standardized TOCS *z* scores. Total scores were modeled using linear regression controlling for age and gender, for parent- and self-respondents separately and residual scores were obtained. Participants were divided into 30 groups according to respondent (parent- or self-report), gender, and integer age. Parent-report groups included integer ages from 6 to 15 and self-report groups included integer ages from 13 to 17. Standardized scores corresponding to the empirical percentile of each individual were assigned within each of the 30 groups separately. We compared the heritability of the TOCS total score to an established measure of OC symptoms: the CBCL-OCS^22,23^. Each of the eight CBCL-OCS items were scored on a scale of 0–2 (0 = not true; 1 = somewhat/sometimes true; and 2 = very/often true), and were summed to generate a total score (range: 0–16).

### Twin sub-sample

We estimated heritability from 220 twin pairs. Their zygosity was initially determined by a twin questionnaire adapted from Cohen et al.^25^, and confirmed using a 16 marker microsatellite panel following the protocol outlined by Yang et al.^26^ DNA extracted from saliva was analyzed for short tandem repeats using the AmpFLSTR^®^ Identifiler^TM^ PCR Amplification kit (PE Applied Biosystems, Foster City, CA, USA), a panel consisting of 15 autosomal, codominant, unlinked loci, and the sex-determining marker, amelogenin amplified in a single PCR^26^. We classified twin pairs as monozygotic (MZ) if all 16 markers were identical between the pair; otherwise, they were classified as dizygotic (DZ)^26^. We had four sets of DZ triplets. We randomly selected two siblings from each triplet to be a DZ twin pair and excluded the other sibling. Our final twin sample included 60 MZ twin pairs (50% male) and 160 DZ twin pairs (60 male, 33 female, and 67 opposite-sex pairs). The mean age of the twins was 10.5 years (SD 2.6) and no individuals had a reported diagnosis of OCD.

### Statistical analysis

#### Factor analysis

Exploratory factor analysis with principal components using varimax rotation was conducted in SAS 9.3 to examine the underlying dimensional structure of the TOCS. We also conducted promax rotation because of the expected correlation of TOCS items. Phenotypic correlations between the OC trait dimensions were examined using Pearson’s correlations with IBM SPSS Statistics 21.0. We assigned items to factors when the factor loading was > 0.7 and had factor loadings < 0.4 for all other factors. We considered scree plots, cumulative variance explained and interpretability when selecting the number of factors.

#### Heritability analyses

##### Univariate models

Intraclass correlations (ICCs) for each trait and across traits were examined within MZ and DZ twins. The heritability of total OC traits and each OC trait dimension was estimated by structural equation modeling with age, sex, and respondent included as covariates using full information maximum likelihood (including pairs with incomplete data (*n* = 3)) in OpenMx^27^. For analyses using standardized *z* scores, age, sex, and respondent (parent or self) covariates were not included in the models because these factors were incorporated during *z* score calculation (see above). Saturated model fit was conducted to test the assumption of equality of means and variances between the MZ and DZ twins^28^. The goodness of fit parameters used to compare twin models were the likelihood-ratio chi-square statistic (*χ*^2^) and Akaike’s information criterion (AIC).

We decomposed the total variance of the CBCL-OCS, TOCS total scores, and each of the TOCS OC trait dimensions identified in our factor analysis into genetic and environmental factors. Genetic variance could be attributable to additive effects (A), and/or dominance (non-additive) effects (D). Environmental variance was partitioned into common environmental (C) influences, which are shared by family members, and unique environmental (E) factors, which include measurement error. The within-pair additive genetic correlation (A) was set at 1 for MZ twins and 0.5 for DZ twins, whereas the dominance genetic correlation (D) was fixed at 1 for MZ twins and 0.25 for DZ twins^29^. The significance of the individual variance components was assessed by comparing the fit of the full models (ACE and ADE) to the nested sub-models (AE, CE, and E).

To examine sex differences in heritability, we observed intra-pair correlations by zygosity and sex. Our sample only had 33 DZ female twin pairs, and because the opposite-sex DZ twin correlations (0.40) were generally similar to the DZ same-sex twin correlations (males = 0.47; females = 0.41) we did not further test sex differences in heritability. We could not examine differences in heritability by respondent because there were very few self-reporting twins (*n* = 54).

##### Multivariate models

We tested the degree to which A, C, and E factors accounted for the co-variance between the OC trait dimensions for the TOCS. We fit a multivariate correlated factor model to test the assumption that each pair of OC trait dimensions is directly influenced by genetic (A) and environmental (C and E) variance components that are correlated. A correlation between the A variance components of two OC trait dimensions was interpreted as an indication of a shared genetic influence (i.e., the genetic factors that explain variation in the first OC trait dimension explain a proportion of the variation in the second OC trait dimension). A correlation between the C or E variance components was interpreted as an indication of overlapping environmental influences (i.e., the environmental factors that explain variation in the first OC trait dimension explain a proportion of the variation in the second OC trait dimension). A genetic or environmental correlation of zero between two OC trait dimensions would indicate independence in the variance components explaining the variation in those two dimensions.

To understand how A, C, and E factors influence the co-variance between all trait dimensions, we compared the correlated factor model (which examines correlations between each pair of dimensions) to the common and independent pathway models (which consider the correlations between all dimensions simultaneously)^30^. In the common pathway model, the co-variance of the OC trait dimensions is accounted for by a single latent phenotype influenced by shared additive genetic (Ac), common environment (Cc), and unique environment (Ec) factors. The model estimates dimension-specific genetic (As), common environment (Cs), and unique environment factors (Es). The independent pathway model accounts for co-variance of the dimensions by estimating Ac, Cc, and Ec factors that directly influence each dimension (i.e., not through a latent phenotype) and dimension-specific variance is accounted by estimating As, Cs, and Es factors for each dimension. The best fitting model was selected using the AIC.

## Results

### Factor structure

We selected a six-factor structure as it minimized cross-loading between factors while including as many items as possible, accounted for the most variance (75%), and produced the same factor structure for both parent- and self-reported data (an asset for use in population-based samples). Two items, “experiences unwanted upsetting thoughts or images” (referred to as ‘upsetting’) and “spends time checking and rechecking homework” (referred to as “homework”), factored separately from the other items and were excluded from the final factor model. Upon re-examination of these two items, we considered the ‘upsetting” item too general, capturing a broad, non-specific trait, and the “homework” item, intended to capture a checking compulsion, too specific. The six factors were as follows: Cleaning/Contamination, Symmetry/Ordering, Superstition, Rumination, Counting/Checking, and Hoarding (Table 1). Distributions of these factors, as reported in Park et al.^21^, are shown in Supplemental Fig. 1. The results were similar when analyzed using promax and varimax rotation.

**Table 1:** Factor analysis of the TOCS (19 items)

	Factor loading					
TOCS Item	Factor 1: Cleaning/Contamination	Factor 2: Symmetry/Ordering	Factor 3: Superstition	Factor 4: Rumination	Factor 5: Counting/Checking	Factor 6: Hoarding
Wash	0.84	0.09	0.09	0.13	0.19	0.06
Germs	0.84	0.17	0.18	0.10	0.09	0.10
Clean	0.80	0.13	0.17	0.15	0.17	0.05
Dirt	0.77	0.31	0.09	0.11	0.10	0.03
Ruined	0.49	0.40	0.37	0.01	0.07	0.25
Interfere	0.21	0.76	0.17	0.15	0.18	0.21
Not exactly	0.28	0.66	0.19	0.42	0.11	0.16
Symmetrical	0.26	0.64	0.12	0.11	0.46	0.10
Repeat	0.24	0.60	0.29	0.24	0.35	0.13
Bad luck	0.17	0.18	0.79	0.08	0.27	0.21
Special	0.14	0.25	0.74	0.11	0.34	0.05
Healthy	0.35	0.11	0.64	0.33	0.07	0.17
Guilty	0.17	0.19	0.14	0.82	0.25	0.11
Thinking	0.18	0.23	0.17	0.80	0.27	0.12
Checks	0.27	0.12	0.17	0.25	0.74	0.07
Count	0.17	0.40	0.26	0.15	0.66	0.15
Do certain	0.09	0.20	0.26	0.22	0.71	0.15
Throwing	0.12	0.15	0.12	0.15	0.09	0.87
Useless	0.07	0.17	0.16	0.07	0.16	0.87

The table shows factor loadings for each of the 19 items on the six obsessive–compulsive (OC) dimensions from the Toronto Obsessive–Compulsive Scale (TOCS)

Phenotypic inter-factor correlations are shown in Table 2. The highest correlation was observed between Counting/Checking and Symmetry/Ordering (*r* = 0.70). Hoarding was less correlated with the other five dimensions (*r* = 0.31–0.52) and the TOCS total score (*r* = 0.57; Table 2).

**Table 2:** Factor–factor phenotypic correlations

	OC trait dimensions										
	Cleaning/Contamination		Symmetry/Ordering		Superstition		Rumination		Counting/Checking		Hoarding
Cleaning/Contamination	1		−		−		−		−		−
Symmetry/Ordering	0.62	***0.59***	1		−		−		−		−
Superstition	0.55	***0.58***	0.62	***0.65***	1		−		−		−
Rumination	0.44	***0.45***	0.60	***0.64***	0.51	***0.59***	1		−		−
Counting/Checking	0.50	***0.52***	0.70	***0.72***	0.63	***0.70***	0.60	***0.66***	1		−
Hoarding	0.31	***0.29***	0.45	***0.52***	0.42	***0.47***	0.34	***0.49***	0.52	***0.56***	1
TOCS Total Score	0.80		0.88		0.79		0.73		0.81		0.57
*p* ≤ 0.01 for all values											

Pearson’s correlation values between each of the six obsessive–compulsive (OC) trait dimensions. The values on the left show correlations in the whole sample (*n* = 16,718), and the bold, italicized values show correlations from the twin sub-sample (*n* = 220 pairs). TOCS = Toronto Obsessive–Compulsive Scale

We also considered an alternative model with four factors. Details of this model, the phenotype correlations (Supplemental Table 2) and heritability results (Supplemental Table 3–5, Supplemental Figure S3) for the four-factor model are in the supplemental material.

### Univariate heritability models

ICCs for the TOCS total and dimension scores in the twins are shown in Table 3. No differences in the means and variances for the MZ and DZ twins were observed. For all variables, ICCs were larger for MZ than DZ twins, suggesting a genetic contribution to OC traits. MZ twin correlations were about double the DZ correlations across traits except for Cleaning/Contamination (Supplemental Table 6). Table 3 provides the standardized parameter estimates for the ACE or AE models. The small sample size resulted in low power to detect small effects. For example, heritability of the Cleaning/Contamination dimension was 30%, which is not negligible, but was not statistically significant (95% confidence interval: 0.0–0.66).

**Table 3:** Univariate heritability analyses of overall OC trait and dimensions

Variable	ICC		Best fitting model	Δ AIC	Δ *χ*^2^	Δ *df*	*p* -value	A (CI)	C (CI)	E (CI)
	MZ (CI) (*N*=120)	DZ (CI) (*N*=320)								
TOCS										
Total *Z* score	0.74 (0.60–0.83)	0.37 (0.23–0.50)	AE	7.48	6.52	7	0.48	0.74 (0.63, 0.82)	n/a	0.26 (0.18, 0.37)
CBCL-OCS										
Total score	0.62 (0.44–0.75)	0.34 (0.20–0.47)	AE	4.95	9.05	7	0.25	0.56 (0.40, 0.68)	n/a	0.44 (0.32, 0.60)
TOCS dimensions										
Cleaning/Contamination	0.56 (0.36–0.71)	0.40 (0.27–0.53)	ACE	7.60	4.40	6	0.62	0.30 (0, 0.66)	0.26 (0, 0.52)	0.45 (0.31, 0.63)
Symmetry/Ordering	0.72 (0.57–0.82)	0.30 (0.15–0.43)	AE	8.67	5.33	7	0.62	0.70 (0.57, 0.79)	n/a	0.30 (0.21, 0.43)
Superstition	0.70 (0.54–0.81)	0.46 (0.33–0.58)	ACE	10.38	1.62	6	0.95	0.50 (0.17, 0.78)	0.20 (0, 0.44)	0.29 (0.20, 0.43)
Rumination	0.55 (0.35–0.71)	0.27 (0.12–0.41)	AE	6.04	7.97	7	0.34	0.53 (0.36, 0.66)	n/a	0.47 (0.34, 0.64)
Counting/Checking	0.76 (0.62–0.85)	0.37 (0.23–0.50)	AE	7.22	6.78	7	0.45	0.77 (0.66, 0.84)	n/a	0.23 (0.16, 0.34)
Hoarding	0.66 (0.49–0.78)	0.31 (0.16–0.44)	AE	4.54	9.46	7	0.22	0.61 (0.46, 0.72)	n/a	0.39 (0.28, 0.54)

The table shows intraclass correlations (ICC) within the monozygotic (MZ) and dizygotic (DZ) twins followed by Akaike’s information criterion (Δ AIC) differences, chi-square (Δ*χ*2) differences, degrees of freedom (Δ*df*) differences, and the *p* values comparing the saturated model to the ACE model. 95% confidence intervals (CI) are shown. A: additive genetic influence; C: common environmental influence; D: non-additive genetic (or dominance) influence; E: unique environmental influence. *OC* obsessive–compulsive, *TOCS* Toronto Obsessive–Compulsive Scale, *CBCL-OCS* the Obsessive–Compulsive Scale of the Child Behavior Checklist

Heritability of OC traits was based on the estimates for TOCS *z* score and CBCL-OCS total score. Additive genetic factors accounted for 74% of the variance of OC traits measured by the TOCS *z* score with 26% of the variance explained by unique environmental factors. For the CBCL-OCS, the genetic contribution was 56%, with unique environmental accounting for the remaining variance.

As shown in Table 3, the AE model fit well for most of the dimensions, although the ACE model was the most parsimonious for the Cleaning/Contamination and Superstition dimensions based on AIC. Considerable genetic contributions were observed for all dimensions with heritability estimates ranging from 30 to 77%. At least half of the variance of the dimensions was explained by genetics with the exception of Cleaning/Contamination, where approximately 70% of its variance was explained by environment, with 26% explained by common environment.

### Multivariate heritability models

We examined the genetic correlations of the OC dimensions in our twin sample by decomposing the co-variance between pairs of dimensions into genetic and environmental components to estimate the extent that these components influenced the dimensions (Table 4).

**Table 4:** Multivariate twin analysis matrices for all OC dimensions

	Cleaning/Contamination	Symmetry/Ordering	Superstition	Rumination	Counting/Checking	Hoarding
Additive genetic influence (A) correlations with 95% CI						
Cleaning/Contamination		-	-	-	-	-
Symmetry/Ordering	0.58 (0.08, 1)		-	-	-	-
Superstition	0.60 (− 0.1, 0.99)	0.70 (0.41, 0.99)		-	-	-
Rumination	0.57 (− 0.28, 0.96)	0.86 (0.64, 1)	0.82 (0.35, 1)		-	-
Counting/Checking	0.69 (0.33, 1)	0.86 (0.73, 0.96)	0.82 (0.62, 1)	0.83 (0.60, 1)		-
Hoarding	0.63 (0.05, 1)	0.77 (0.52, 0.96)	0.62 (0.19, 0.90)	0.50 (− 0.02, 1)	0.60 (0.30, 0.87)	
Common environmental influence (C) correlations with 95% CI						
Cleaning/Contamination		-	-	-	-	-
Symmetry/Ordering	0.78 (− 1, 1)		-	-	-	-
Superstition	0.72 (− 0.49, 1)	0.99 (− 1, 1)		-	-	-
Rumination	0.28 (− 1, 1)	0.68 (− 1, 1)	0.65 (− 1, 1)		-	-
Counting/Checking	0.79 (− 1, 1)	1 (− 1, 1)	0.98 (− 1, 1)	0.71 (− 1, 1)		-
Hoarding	0.14 (− 1, 1)	0.72 (− 1, 1)	0.76 (− 1, 1)	0.86 (− 1, 1)	0.72 (− 1, 1)	
Unique environmental influence (E) correlations with 95% CI						
Cleaning/Contamination		-	-	-	-	-
Symmetry/Ordering	0.53 (0.3, 0.67)		-	-	-	-
Superstition	0.47 (0.27, 0.63)	0.43 (0.21, 0.61)		-	-	-
Rumination	0.41 (0.21, 0.58)	0.39 (0.20, 0.56)	0.36 (0.15, 0.55)		-	-
Counting/Checking	0.22 (0, 0.43)	0.36 (0.15, 0.55)	0.35 (0.14, 0.55)	0.46 (0.24, 0.62)		-
Hoarding	0.04 (− 0.17, 0.26)	0.10 (-0.11, 0.32)	0.13 (− 0.09, 0.35)	0.27 (0.05, 0.46)	0.34 (0.11, 0.53)	

Univariate estimates for each dimension not shown

The table shows the correlations of additive genetic (A), common environmental (C), and unique environmental (E) variance between each of the obsessive–compulsive (OC) dimensions with 95% confidence intervals (CI)

Additive genetic correlations between OC trait dimensions accounted for the majority of their co-variance. Significant correlations between A were observed for most pairs of dimensions except for Cleaning/Contamination with Superstition and Rumination. The highest additive genetic correlation was observed for Symmetry/Ordering and Counting/Checking (0.86) and for Symmetry/Ordering and Rumination (0.86). Unique environmental influences also accounted for significant co-variance between OC trait dimensions. The Cleaning/Contamination and the Symmetry/Ordering dimensions showed highest E correlations (0.53). The lowest unique environmental correlation was for the Cleaning/Contamination and Hoarding dimension (0.04).

**Fig. 1:**
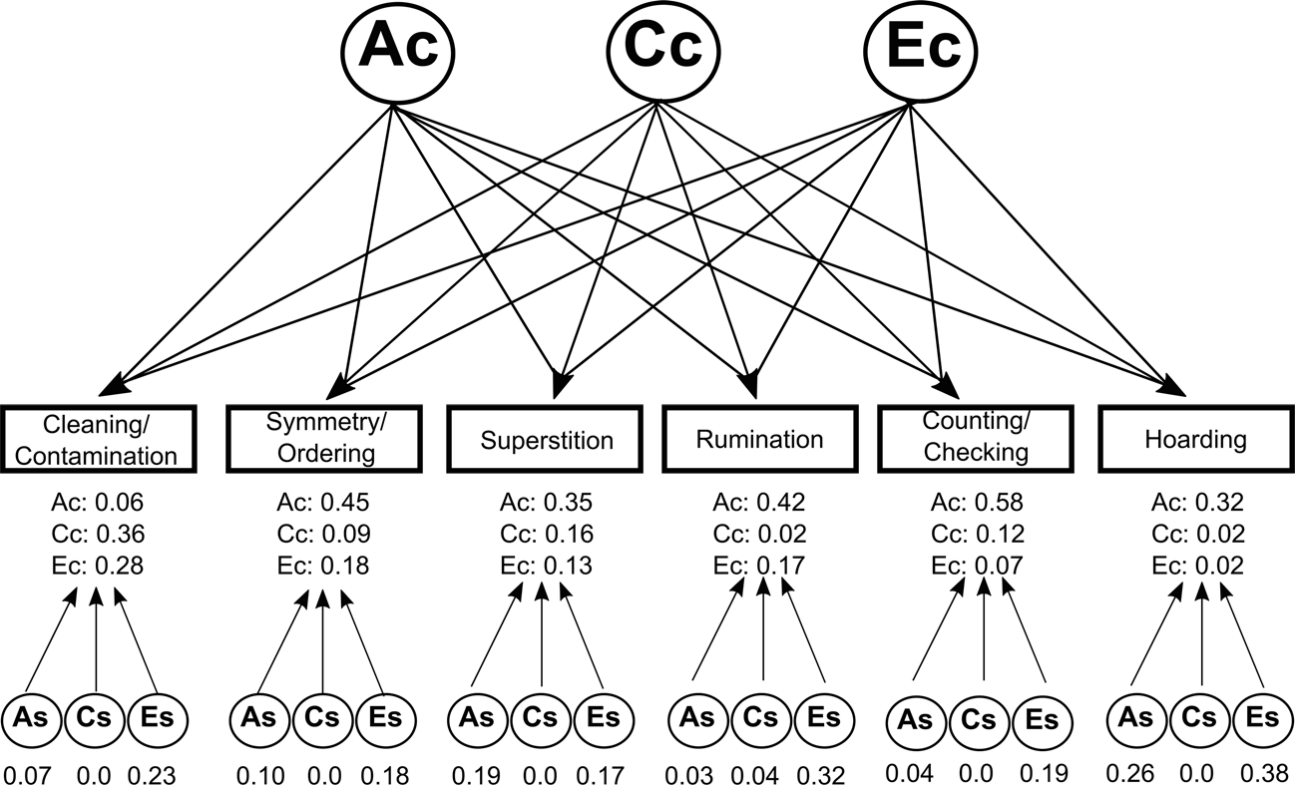
Independent pathway model of OC dimensions. The independent pathway model afforded the optimal fit to the obsessive–compulsive (OC) dimension data. Shared variance was mostly attributed to shared additive genetic influences (Ac), whereas dimension-specific variance was mostly explained by unique environment (Es). Additive genetic factors (As) accounted for dimension-specific variance for Hoarding and Superstition. Shared (Cc) and dimension-specific (Cs) common environment only explained considerable variance for Cleaning/Contamination

We compared the fit of the ACE common pathway (AIC = 8259.76, *df* = 2604, *p* *=* 0.02) and independent pathway (AIC = 8243.88, *df* = 2595, *p* *=* 0.22) models to the correlated factor model (AIC = 8265.35, *df* = 2568) for the OC trait dimensions. The independent pathway model was the most parsimonious. Model fit was unchanged by removing the Hoarding dimension (data not shown). As shown in Fig. 1, the majority of shared variance for each dimension was accounted for by genetic factors (Ac = 32–58%), except Cleaning/Contamination where common environment (Cc) accounted for the majority of the variance (36%). Genetic influences (As) accounted for the majority of dimension-specific variance only for Hoarding and Superstition (19–26%). For all other dimensions, unique environment influences (Es) accounted for the majority of the dimension-specific variance (Es = 17–38%). Variance estimates from the independent model are presented in Supplemental Table 4.

## Discussion

One strategy for improving the power of genetic studies in OCD is to focus on OC traits rather than on OCD diagnoses and to measure these traits in a way that generates widely distributed scores in the general population. The added variance and scope of behaviors captured by these trait-based measures may improve power and reduce error in genetic studies. We developed the TOCS to measure the full range of OC traits in youth^21^. To be informative for genetic research of OCD, the TOCS should be heritable and so should any OC trait dimensions that it captures. The TOCS factored into six heritable and co-heritable OC dimensions similar to those reported from studies using existing OCD scales^13,14^. We used ACE twin models to compare independent and common pathway models and to assess the degree to which dimensions shared etiological factors. Using twin models, we found statistically significant heritability and co-heritability of the TOCS total score and individual TOCS dimensions. However, power for detecting sex or age differences was limited. We showed that the genetic and environmental influences on the dimensions were best explained by an independent model and not by a latent trait. TOCS dimensions exhibited both shared and unique genetic influences. Hoarding was less phenotypically correlated with the other dimensions but was still genetically correlated and shared part of its additive genetic influences with other OC dimensions. Although Hoarding did standout phenotypically, it shared a common underlying etiology with other OC dimensions. Together our results show that the TOCS is an informative measure for genetic research when used either as a single global OC trait or as individual dimensions.

Overall, there was considerable convergence in the factor structure of the TOCS and existing OCD scales^13,14,31–33^. One important difference from previous studies is that we identified separate Counting/Checking and Rumination dimensions, which often cluster with other OC symptoms. Counting and Checking symptoms often cluster with Symmetry/Ordering and/or Hoarding^13^. The Rumination dimension contained items consistent with symptoms from the Sexual/Religious obsession dimension (symptoms not queried on the TOCS) from previous studies^17,32,34^.

The estimated heritability of the TOCS total score was 74%, which was higher than estimates for OC symptoms from previous twin studies using existing OC measures^6,8,9,35–37^. The TOCS also had higher heritability estimates than the CBCL-OCS, an established heritable OC measure^9^, supporting the utility of the TOCS in genetic research. Each OC trait dimension was heritable, a finding that converges with previous studies of youth and adults^15–17,36,38,39^. Environmental factors contributed significantly to only the Cleaning/Contamination dimension, suggesting a distinct etiological mechanism. This finding is consistent with a previous study in an adolescent population-based sample that also reported low additive genetic effects for this dimension^17^. The effect of common environment on Cleaning/Contamination may result from family values, education or parental modeling.

Phenotypic heterogeneity in the TOCS, demonstrated by six OC dimensions, also reflected some etiological heterogeneity. Genetic factors contributed considerably to all OC dimensions, although less so for Cleaning/Contamination. All OC dimensions were also co-heritable indicating that they share some genetic influences. However, the degree to which genetic effects influenced the variance shared by dimensions (Ac) rather than the dimensions individually (As) varied by dimension. For many OC dimensions, shared effects (Ac) accounted for genetic influences with the other dimensions, suggesting that similar genetic factors played an important role across phenotypically separate, but correlated, OC dimensions. By contrast, what made the OC dimensions different was accounted for mostly by unique environment (Es) rather than genetic factors (As). A notable exception was Hoarding that had considerable genetic effects that were shared with the other dimensions (Ac) but also had considerable Hoarding-specific genetic influences (As). A similar pattern was observed for Superstition. Unlike our study, a previous study of female adults^15^ reported dimension-specific genetic effects for most OC dimensions. However, both ours and this previous study reported that unique environment accounted for most of the variance specific to each dimension (Es), suggesting that environment and potentially error (which is not separable in ACE models) are the biggest contributors to making dimensions different. One implication is that genetics may play a larger role in what makes dimensions similar, whereas unique environment may play a bigger role in what makes dimensions different.

The finding that an independent model best explained the shared variance between dimensions highlights both shared and unique etiological influences. If a common pathway model had fit best, shared etiology of the OC dimensions would have been attributable to a latent trait (e.g., OC traits). If that were true, it would be necessary to calculate a latent trait for genetic studies rather than studying individual dimensions. OC dimensions can be highly correlated and still fit an independent model. Both independent and common models demonstrate that the co-variance between dimensions can be accounted for in part by shared genetic factors. However, in the independent model, those shared genetic factors influence each dimension directly, whereas in the common model these shared genetic factors are so closely related that they influence each dimension through a common factor. Results from previous studies in adults on the fit of the common and independent pathway for OC traits are mixed^15,16^. An important difference seems to be the number of OC dimensions captured by the scale they used—an independent model fits best when there were more dimensions^15^, whereas a common model fit best when there were fewer dimensions^16^. Our finding that shared etiological factors contribute to OC dimensions in youth without being mediated by a latent trait suggests that simply measuring overall OC traits will not uncover the full spectrum of genetic influences on OC dimensions and that OC traits are heterogeneous.

In the DSM-5, hoarding is considered both a possible symptom of OCD and a symptom of a distinct hoarding disorder^18^. In our study, Hoarding did not correlate as well phenotypically with the other dimensions or the TOCS total score but was genetically correlated with all other OC trait dimensions. Hoarding also differed from the other dimensions in that genetic influences contributed significantly to both shared and dimension-specific variance. Excluding Hoarding from the independent pathway model did not affect model fit, suggesting that Hoarding was not obscuring a latent OC trait that accounted for the other OC dimensions. A study of adult twins also showed that hoarding had both specific and unique genetic effects and that an independent model fit the data best with or without the hoarding dimension^15^. In another previous adult twin study, hoarding and total OC symptoms were not highly genetically correlated but did share additive genetic effects^20^. Probable hoarding disorder and OC symptoms also shared genetic effects in another sample of adult twins^19^. Classifying hoarding as a distinct condition is useful in the clinic and may help in identifying hoarding-specific mechanisms. However, our results and others indicate hoarding shares considerable genetic risk with other OC trait dimensions. Disorders may share their genetic etiology even when phenotypically and clinically distinct^40^.

We conclude that the TOCS is useful for studying both an overall OC trait and individual dimensions. This measure identified several heritable OC dimensions similar to those in previous studies. OC dimensions were correlated to a significant extent, but appeared to have different etiological mechanisms. Even less phenotypically correlated dimensions shared genetic risk. We are currently working on a genome-wide association study of OC traits in this sample to uncover whether genetic variants are associated similarly across OC dimensions. Specifically, we will test if Hoarding and Cleaning/Contamination are differentially associated with genetic variants. To uncover the genetics of OC traits and OCD, OC trait dimensions should be considered both individually and together.

## Electronic supplementary material

Supplemental Material
