# Supplementary material for: Heritability of obsessive–compulsive trait dimensions in youth from the general population
Source: Transl Psychiatry. 2018 Sep 18;8:191. doi: 10.1038/s41398-018-0249-9 (PMC6143601; doi:10.1038/s41398-018-0249-9)

Supplemental Text

Alternative Factor Structure

The factor structure revealed OC traits were dimensional, however, more than one factor structure could fit our data depending on the criteria used. We considered a four-factor solution based on a scree plot with a minimum eigenvalue of 1 (Supplemental Figure 2). However, several items loaded strongly onto more than one factor with secondary factor loadings > 0.5 (see Supplemental Table 1). These items queried common OC behaviours (e.g., symmetry, repeating, etc.) so we did not exclude these items from the factor analysis to include as many relevant items as possible. We found that six factors reduced the cross-loading, although did not eliminate it (see Table 1 in main text). We also considered a six-factor model with only 14 items, which again removed common OC behaviours, so these items were not excluded. In both the four- and six-factor structures there were clear Cleaning/Contamination and Hoarding factors. In the four-factor model, Symmetry/Ordering factored with Rumination and Superstition factored with Counting. In the six-factor model, these were four separate dimensions.

The six-factor structure model was chosen because it minimized cross-loading across factors while still including common OC symptoms such as repeating and counting. The six-factor structure resulted in the same dimensions in both parent- and self-ratings optimizing power in population based studies of youth by allowing the use of a single measure across informants.

We also examined the heritability of the four trait dimensions. All of the dimensions were heritable and the AE model fit each dimension except Cleaning/Contamination where the ACE was the most parsimonious model (Supplemental Table 3). The genetic correlations were also similar for the four- and six-factor model (Supplemental Table 4). An independent model fit the

data best (AIC = 6633.20, df = 1729, $p = 0.82$) compared to the common pathway (AIC = 6643.98, df = 1734, $p = 0.01$) and correlated factor models (AIC = 6642.28, df = 1723). Genetic effects (Ac) still accounted for the majority of the shared variance between dimensions (39-53%) except for Cleaning/Contamination where shared environment (Cc) accounted for the most variance. Genetic influences (As) still accounted for a significant proportion of dimension-specific variance for hoarding as seen in the six-factor model. One difference was that genetic effects now also accounted for more dimension-specific variance than with the six-factor model. See Supplemental Figure 3 and Table 5 for details. We also compared the heritability of the six dimensions with the initial factor structure using 14 items and the factor structure using 19 items that included more clinically-relevant items. The univariate and multivariate heritability results were similar to the six-factor structure with 19 or 14 items (data not shown).

An important finding from our results is that regardless of the number of factors included or the number of items included, our genetic results were similar for the heritability and co-heritability of the dimensions in that genetic effects accounted for considerable variance for each dimension. The independent model fit the data best and the shared and dimension-specific variance estimates were similar for most dimensions. One notable difference was that for Cleaning/Contamination, the proportion of shared and dimension-specific variance accounted for by genetic and common environment fluctuated based on the factor structure/number of items. This finding suggests that the relative contribution of genetic and common environmental effects is less clear for Cleaning/Contamination, a conclusion that is supported by our univariate models and previous literature[1, 2]. The fact that almost all of the heritability results were similar regardless of factor structure suggests that the heritability and co-heritability of OC dimensions is robust.

References

1. Moore J, Smith GW, Shevlin M, O'Neill FA. Alternative factor models and heritability of the Short Leyton Obsessional Inventory-Children's Version. *J Abnorm Child Psychol* 2010; **38**(7)**:** 921-934.

2. Iervolino AC, Rijsdijk FV, Cherkas L, Fullana MA, Mataix-Cols D. A multivariate twin study of obsessive-compulsive symptom dimensions. *Arch Gen Psych* 2011; **68**(6)**:** 637-644.

Supplemental Table 1: Factor analysis of the TOCS (four-factor model). The table shows factor loadings for each of the 19 items on the four obsessive-compulsive (OC) dimensions from the Toronto Obsessive-Compulsive Scale (TOCS).

	Factor Loading			
**TOCS Item**	**Factor 1: Cleaning/ Contamination**	**Factor 2: Symmetry/ Ordering/Rumination**	**Factor 3: Superstition/Counting**	**Factor 6: Hoarding**
Wash	0.82	0.20	0.14	0.03
Germs	0.85	0.16	0.14	0.03
Clean	0.80	0.22	0.19	0.04
Dirt	0.80	0.25	0.13	0.06
Ruined	0.55	0.14	0.39	0.33
Thinking	0.18	0.81	0.16	0.13
Guilty	0.17	0.80	0.11	0.11
Not Exactly	0.36	0.63	0.22	0.27
Repeat	0.30	0.55	0.46	0.20
Symmetrical	0.30	0.54	0.40	0.15
Checks	0.22	0.52	0.48	-0.01
Interfere	0.30	0.49	0.31	0.33
Bad Luck	0.22	0.12	0.80	0.25
Special	0.18	0.21	0.80	0.10
Count	0.18	0.52	0.58	0.13
Do Certain	0.06	0.51	0.56	0.10
Healthy	0.38	0.23	0.50	0.21
Throwing	0.11	0.19	0.12	0.87
Useless	0.07	0.16	0.21	0.86

Supplemental Table 2: Factor-Factor Phenotypic Correlations (four-factor model).

Pearson's correlation values between each of the four obsessive-compulsive (OC) trait dimensions. The values on the left show correlations in the whole sample (n=16,718), and the bold values show correlations from the twin sub-sample (n = 220 pairs).

	**OC Trait Dimensions**							
	Cleaning/ Contamination		Symmetry/ Ordering/Rumination		Superstition/Counting		Hoarding	
Cleaning/ Contamination	1		-		-		-	
Symmetry/ Ordering/Rumination	0.62	***0.60***	1		-		-	
Superstition/Counting	0.57	***0.58***	0.77	***0.80***	1		-	
Hoarding	0.31	***0.30***	0.45	***0.54***	0.45	***0.53***	1	
TOCS Total Score	0.80		0.93		0.87		0.57	
$p \leq 0.01$ for all values								

Supplemental Table 3: Univariate Variance Estimates for OC Dimensions (four-factor model) The table shows intraclass correlations (ICC) within the monozygotic (MZ) and dizygotic (DZ) twins followed by Akaike's information criterion (Δ AIC) differences, chi-square (Δ χ^2) differences, degrees of freedom (Δ df) differences, and the p-values comparing the saturated model to the ACE model. 95% confidence intervals (CI) are shown. A: additive genetic influence; C: common environmental influence; D: non-additive genetic (or dominance) influence; E: non-shared environmental influence; TOCS= Toronto Obsessive-Compulsive Scale.

Variable	ICC		Best Fitting model	Δ AIC	Δ $\chi 2$	Δ df	p-value	A (CI)	C (CI)	E (CI)
	MZ (CI) (N=120)	DZ (CI) (N=320)								
**TOCS Dimensions**										
Cleaning/ Contamination	0.56 (0.36-0.71)	0.40 (0.27-0.53)	ACE	7.60	4.40	6	0.62	0.30 (0, 0.66)	0.26 (0, 0.52)	0.45 (0.31, 0.63)
Symmetry/ Ordering/Rumination	0.74 (0.57-0.82)	0.34 (0.19-0.47)	AE	4.54	9.46	7	0.22	0.72 (0.60, 0.81)	n/a	0.28 (0.19, 0.40)
Superstition/Counting	0.78 (0.65-0.86)	0.34 (0.19-0.47)	AE	8.16	5.83	7	0.56	0.50 (0.17, 0.78)	0.20 (0, 0.44)	0.29 (0.20, 0.43)
Hoarding	0.66 (0.49-0.78)	0.31 (0.16-0.44)	AE	4.54	9.46	7	0.22	0.79 (0.69, 0.85)	n/a	0.21 (0.15, 0.31)

Supplemental Table 4. Multivariate Twin Analysis Matrices for all OC Dimensions (four-factor model). The table shows the correlations of additive genetic (A), common environmental (C) and unique environmental (E) variance between each of the obsessive-compulsive (OC) dimensions with their 95% confidence intervals (CI).

	Cleaning/ Contamination	Symmetry/ Ordering/Rumination	Superstition/Counting	Hoarding
**Additive Genetic Influence (A) Correlations with 95% CI**				
Cleaning/ Contamination		-	-	-
Symmetry/ Ordering/Rumination	0.66 (0.0, 1)		-	-
Superstition/Counting	0.57 (0, 1)	0.87 (0.73, 0.99)		-
Hoarding	0.70 (-0.01, 1)	0.75 (0.46, 0.98)	0.74 (0.43, 1)	
**Common Environmental Influence (C) Correlations with 95% CI**				
Cleaning/ Contamination		-	-	-
Symmetry/ Ordering/Rumination	0.75 (-1, 1)		-	-
Superstition/Counting	0.87 (0, 1)	0.98 (-1, 1)		-
Hoarding	0.21 (-1, 1)	0.80 (-1, 1)	0.67 (-1, 1)	
**Unique Environmental Influence (E) Correlations with 95% CI**				
Cleaning/ Contamination		-	-	-
Symmetry/ Ordering/Rumination	0.56 (0.36, 0.70)		-	-
Superstition/Counting	0.46 (0.25, 0.63)	0.60 (0.42, 0.74)		-
Hoarding	0.04 (-0.18, 0.26)	0.23 (0.01, 0.44)	0.20 (-0.03, 0.42)	

Supplemental Table 5. Best Fitting Multivariate Model: Independent Pathway (four-factor model). The independent pathway model fit the multivariate obsessive-compulsive (OC) dimensions best. Variance estimates including 95% confidence intervals (CI) are shown for the shared additive genetic (Ac), common environment (Cc) and unique environment (Ec) that directly influence each dimension and dimension-specific additive genetic (As), common environment (Cs) and unique environment (Es).

	Total variance (95% CI)			Common variance (95% CI)			Dimension-Specific variance (95% CI)		
	A	C	E	Ac	Cc	Ec	As	Cs	Es
Cleaning/ Contamination	0.24 (0.01-0.52)	0.30 (0.07-0.51)	0.46 (0.34-0.63)	0.09 (0.01-0.30)	0.30 (0.07-0.51)	0.20 (0.09-0.36)	0.15 (0.01-0.34)	0 (0-0)	0.26 (0.17-0.39)
Symmetry/Ordering/ Rumination	0.59 (0.35-0.75)	0.09 (0.01-0.28)	0.32 (0.22-0.45)	0.53 (0.25-0.71)	0.09 (0.01-0.28)	0.25 (0.15-0.38)	0.06 (0.01-0.014)	0 (0-0)	0.07 (0.01-0.15)
Superstition/ Counting	0.61 (0.35-0.81)	0.16 (0.01-0.38)	0.23 (0.16-0.33)	0.51 (0.25-0.73)	0.16 (0.01-0.38)	0.11 (0.05-0.20)	0.10 (0.01-0.19)	0 (0-0)	0.12 (0.07-0.18)
Hoarding	0.58 (0.37-0.70)	0.01 (0.01-0.16)	0.41 (0.30-0.55)	0.39 (0.23-0.53)	0.01 (0.01-0.13)	0.02 (0.01-0.08)	0.19 (0.01-0.33)	0 (0-0.13)	0.39 (0.29-0.52)

Supplemental Table 6: Cross-Twin and Cross-Trait Correlations (six-factor model)

Pearson's correlations within each of the six obsessive-compulsive (OC) trait dimensions (grey cells) and across the OC trait dimensions with the 95% confidence intervals (CI). MZ = monozygotic twins, DZ = dizygotic twins.

		Cleaning/ Contamination	Symmetry/ Ordering	Superstition	Rumination	Counting/ Checking	Hoarding
Cleaning/ Contamination	MZ	0.56 (0.36;0.71)					
	DZ	0.40 (0.27;0.53)					
Symmetry/ Ordering	MZ	0.40 (0.17;0.59)	0.72 (0.57;0.82)				
	DZ	0.22 (0.07;0.36)	0.30 (0.15;0.43)				
Superstition	MZ	0.33 (0.09;0.54)	0.56 (0.35;0.71)	0.70 (0.54;0.81)			
	DZ	0.31 (0.16;0.44)	0.35 (0.21;0.48)	0.46 (0.33;0.58)			
Rumination	MZ	0.29 (0.04;0.51)	0.49 (0.27;0.66)	0.51 (0.30;0.68)	0.56 (0.36;0.71)		
	DZ	0.19 (0.03;0.33)	0.22 (0.07;0.37)	0.27 (0.12;0.41)	0.27 (0.12;0.41)		
Counting/ Checking	MZ	0.42 (0.19;0.61)	0.64 (0.47;0.77)	0.60 (0.41;0.74)	0.57 (0.37;0.72)	0.76 (0.62;0.85)	
	DZ	0.33 (0.19;0.47)	0.35 (0.21;0.48)	0.42 (0.28;0.54)	0.28 (0.13;0.42)	0.37 (0.23;0.50)	
Hoarding	MZ	0.14 (-0.11;0.38)	0.52 (0.31;0.68)	0.55 (0.34;0.70)	0.39 (0.15;0.59)	0.47 (0.25;0.65)	0.66 (0.49;0.78)
	DZ	0.12 (-0.04;0.27)	0.21 (0.06;0.35)	0.19 (0.04;0.34)	0.10 (-0.06;0.25)	0.20 (0.05;0.34)	0.31 (0.16;0.44)

Supplemental Table 7. Best Fitting Multivariate Model: Independent Pathway (six-factor model). The independent pathway model fit the multivariate obsessive-compulsive (OC) dimensions best. Variance estimates including 95% confidence intervals (CI) are shown for the shared additive genetic (Ac), common environment (Cc) and unique environment (Ec) that directly influence each dimension and dimension-specific additive genetic (As), common environment (Cs) and unique environment (Es).

	Total variance (95% CI)			Common variance (95% CI)			Dimension-Specific variance (95% CI)		
	**A**	**C**	**E**	**Ac**	**Cc**	**Ec**	**As**	**Cs**	**Es**
Cleaning/Contamination	0.13 (0-0.43)	0.36 (0.14-0.52)	0.51 (0.37-0.66)	0.06 (0-0.21)	0.36 (0.13-0.52)	0.28 (0.16-0.44)	0.07 (0-0.29)	0 (0-0)	0.23 (0.13-0.35)
Symmetry/Ordering	0.55 (0.34-0.70)	0.09 (0-0.25)	0.36 (0.26-0.49)	0.45 (0.25-0.60)	0.09 (0-0.25)	0.18 (0.09-0.29)	0.10 (0-0.19)	0 (0-0)	0.18 (0.11-0.27)
Superstition	0.54 (0.28-0.70)	0.16 (0.03-0.36)	0.30 (0.22-0.42)	0.35 (0.17-0.51)	0.16 (0.03-0.34)	0.13 (0.07-0.22)	0.19 (0-0.28)	0 (0-0)	0.17 (0.11-0.27)
Rumination	0.45 (0.26-0.64)	0.06 (0-0.21)	0.49 (0.35-0.64)	0.42 (0.26-0.55)	0.02 (0-0.12)	0.17 (0.08-0.29)	0.03 (0-0.20)	0.04 (0-0.13)	0.32 (0.21-0.42)
Counting/Checking	0.62 (0.39-0.78)	0.12 (0.01-0.30)	0.26 (0.18-0.37)	0.58 (0.35-0.75)	0.12 (0.01-0.30)	0.07 (0.02-0.16)	0.04 (0-0.12)	0 (0-0.05)	0.19 (0.13-0.27)
Hoarding	0.58 (0.31-0.70)	0.02 (0-0.21)	0.40 (0.29-0.54)	0.32 (0.20-0.44)	0.02 (0-0.13)	0.02 (0-0.08)	0.26 (0-0.39)	0 (0-0.18)	0.38 (0.27-0.51)

Supplemental Figure S1: Distributions of Obsessive-Compulsive Traits

Distribution of the Toronto Obsessive-Compulsive Scale (TOCS) total score (A) and the six OC trait dimensions: Cleaning/Contamination (B), Symmetry/Ordering (C), Superstition, (D), Rumination (E), Counting/Checking (F) and Hoarding (G). A normal distribution was fitted to each distribution. Distributions reproduced with permission from Park et al., 2016.

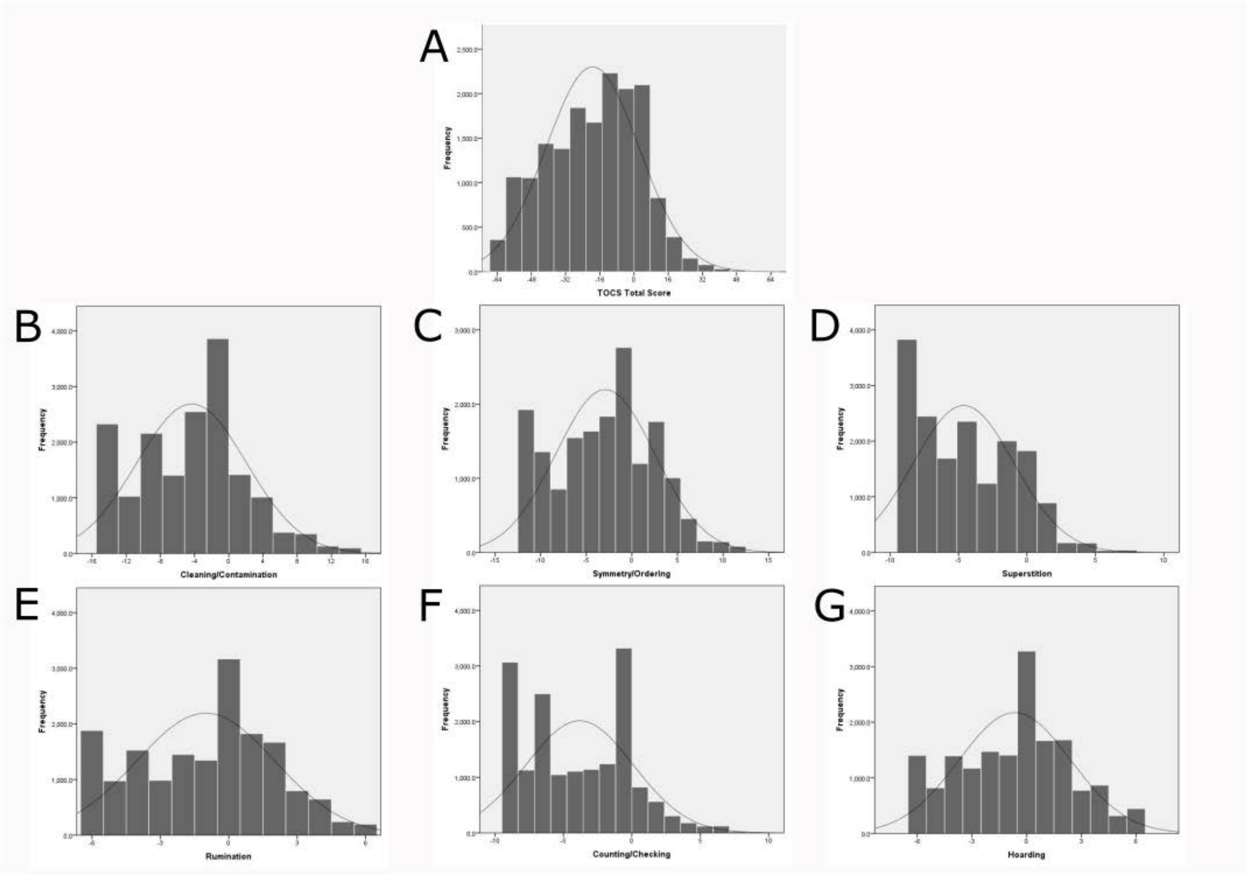

Supplemental Figure S2: Scree Plot.

Scree Plot from factor analysis with 19 items which supports a four-factor model (Eigenvalue = 1.02).

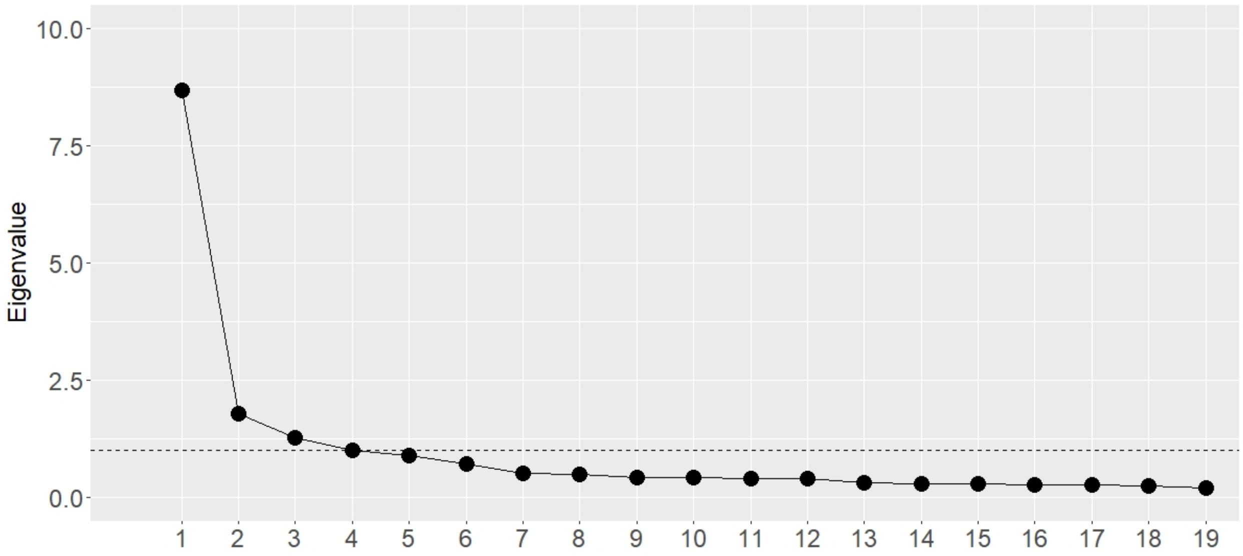

Supplemental Figure S3. Independent Pathway Model (four-factor model).

The independent pathway model fit the obsessive-compulsive (OC) dimension data best. Shared variance was mostly attributed to shared additive genetic influences (Ac) while dimension-specific variance was mostly explained by unique environment (Es). Additive genetic factors (As) accounted for dimension-specific variance for Hoarding and Superstition. Shared (Cc) and dimension-specific (Cs) common environment only explained considerable variance for Cleaning/Contamination.

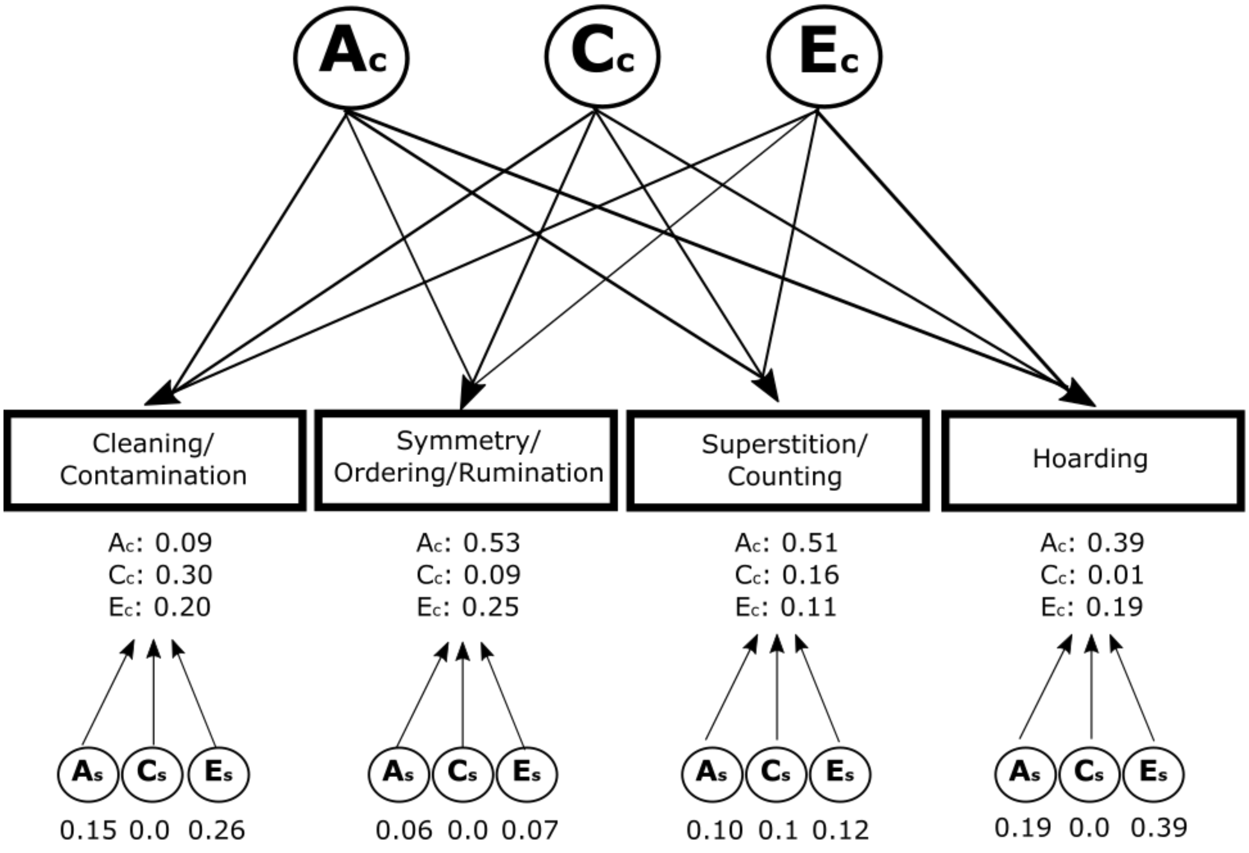

Supplement: Supplementary file 1 — Supplemental Material [file 41398_2018_249_MOESM1_ESM.pdf]
